# Recurrent Large Volume Malignant Pleural Effusion in a Patient With Renal Cell Carcinoma

**DOI:** 10.7759/cureus.13593

**Published:** 2021-02-27

**Authors:** Akil H Hutchinson, Eddie W Fakhouri, Juan Raudales

**Affiliations:** 1 Internal Medicine, NewYork-Presbyterian Brooklyn Methodist Hospital, Brooklyn, USA

**Keywords:** malignant pleural effusions, pleural effusion, renal cell carcinoma

## Abstract

Malignant pleural effusion (MPE) due to renal cell carcinoma (RCC) is extremely rare, accounting for only 1%-2% of all malignant pleural effusions. This paper presents a case report of a 56-year-old male who presented with a chief complaint of bilateral flank pain with dyspnea and was diagnosed with RCC via immunopathologic pleural fluid analysis and who persistently had recurrent large volume pleural effusion.

A 56-year-old male who had a recent admission for dyspnea secondary to a right-sided pleural effusion underwent thoracentesis and returned to the hospital for his worsening shortness of breath. He was found to have recurrent pleural effusion. Thoracentesis studies revealed an exudative pleural effusion positive for malignant cells showing adenocarcinoma, which had an immunopathologic profile (WT-1 and PAX8) favoring an adenocarcinoma of kidney origin. The patient underwent chest tube placement, followed by chemical pleurodesis with 4.3 L of bloody fluid drained immediately. Subsequent x-rays taken while the chest tube was in place showed worsening reaccumulating pleural effusion. A repeat CT scan showed a large right pleural effusion with loculated collections. The patient then underwent right video-assisted thoracoscopic surgery, which revealed a loculated effusion with pleural carcinomatosis that was biopsy-positive for RCC.

This report presents a rare case displaying how RCC pleural carcinomatosis can cause a patient to present with dyspnea secondary to a pleural effusion, which was revealed to be RCC upon fluid cytology and immunohistopathology studies. This case demonstrates that RCC can cause recurrent large volume MPE, which has not been widely reported in contemporary literature.

## Introduction

Renal cell carcinoma (RCC) is the most common type of renal neoplasm, accounting for 85% of renal genitourinary malignancies. Its presentation may be occult, with approximately 10% of cases presenting with weight loss, flank and back pain, uncontrolled hypertension and hematuria [1]. Approximately 50% of cases are incidentally found upon imaging; presentations with pleural effusion are rare, accounting for 1%-2% of malignant pleural effusions [2,3]. RCC lung metastasis is fairly common (50%-60%) and may present as solitary or multiple nodules; however, the presentation of RCC-complicated with malignant pleural effusion (MPE) is rare. Due to the highly vascular nature of RCC, high-volume MPE, as in our case, may be present.

## Case presentation

A 56-year-old male with a history of hypertension, hyperlipidemia, heart failure with reduced ejection fraction 20%, coronary artery disease with two stents one year prior, diabetes mellitus type 2, and a 41-pack-year smoking history presented to the emergency department due to chronic back pain, flank pain, and an outpatient sonogram showing a left-sided renal mass. The patient had been experiencing upper back and flank pain for the past two months, which had not been relieved by over-the-counter pain medications. He unintentionally lost 50 lbs of weight since May 2020.

In the emergency department, his basic metabolic panel was normal with blood urea nitrogen (BUN) 8 mg/dL, creatinine 0.54 mg/dL, glomerular filtration rate >90.0 mL/min/1.73m^2 and urinalysis negative with no hematuria on macroscopic and microscopic analysis. Computed tomographic scan of abdomen/pelvis was obtained showing left-sided kidney mass. The patient was evaluated by Urology, however surgical intervention was deferred due to anticoagulation and risk for bleed. He was admitted for possibility of interventional radiology (IR)-guided biopsy but was postponed due to currently being on ticagrelor and risk of hemorrhage, 

During admission evaluation, further history taking indicated that the patient had been experiencing dyspnea for one week. Chest x-ray showed a right-sided pleural effusion with compressive atelectasis. Thoracentesis was performed, and 2.25 L of serosanguineous fluid was drained (glucose 124 mg/dL, total protein 5.2 mg/dL, lactate dehydrogenase 690 u/L, pH 7.33). The aspirate was bloody, with a white blood cell (WBC) count of 1663/uL (3% neutrophils, 51% lymphocytes, 46% monocytes) and a red blood cell (RBC) count of 203,000/uL. Light’s criteria was suggestive of exudative effusion. The patient’s dyspnea improved and the patient was discharged with a follow-up after one week for cytology results and biopsy of the kidney mass. The patient presented to the emergency room five days later with shortness of breath, and a right-sided Wayne tunneled pleural catheter (TPC) was placed, aspirating 4.3 L of sanguineous fluid. The output from the chest tube gradually decreased, and tissue plasminogen activator (tPA) was inserted.

**Figure 1:**
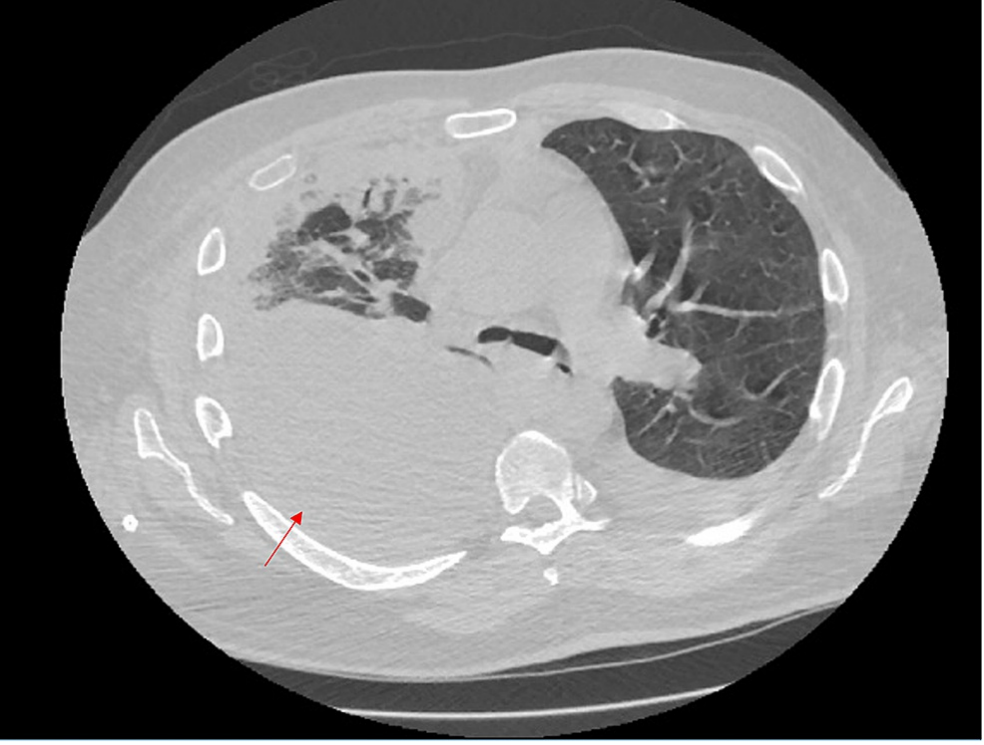
Computed tomographic scan of Chest showing loculated right-sided pleural effusion and left-sided pleural effusion

Although an initial response showed improvement, an intermittent decrease in TPC drainage output was noted. Talc pleurodesis was also performed, but the patient showed no improvement. A subsequent chest CT scan was performed (Figure 1), depicting a large right loculated pleural effusion. Given that his dyspnea was worsening and showed no improvement with a chest tube, video-assisted thoracoscopic surgery (VATS) was performed, confirming a large loculated pleural effusion with pleural carcinomatosis. A second chest tube was subsequently placed. Pleural biopsy was obtained, which later confirmed invasive RCC. IR-guided kidney biopsy was also performed and confirmed RCC. Post-op course was complicated by multiorgan failure with hypoxemic respiratory failure requiring elective intubation and mechanical ventilation for airway protection in light of worsening mental status. 

The patient developed anuric acute tubular necrosis and metabolic acidosis requiring continuous renal replacement therapy. Furthermore, due to worsening hemodynamic instability, three vasopressors (norepinephrine, phenylephrine and vasopressin) were implemented. The patient also underwent direct synchronized cardioversion and amiodarone administration for hemodynamically unstable supraventricular tachycardia. Due to persistent leukocytosis, hypotension, and respiratory acidosis, the patient was started on meropenem and linezolid empirically. The patient continued to have high volume output in chest tubes, which ranged from 700 mL to 2.5 L daily. Despite the interventions, the patient continued to deteriorate and expired during his hospital stay.

## Discussion

Pleural effusions, the accumulation of fluid within the pleural space, may be due to congestive heart failure, pneumonia, malignancy, or pulmonary embolism. With any pleural effusion, hypoxia and dyspnea are often present [4]. A diagnostic and therapeutic aspiration may aid the management of symptoms and the evaluation of the underlying etiology. Diagnostic thoracentesis may involve a small volume of approximately 50 mL and may aid in the differentiation between the types of effusion (i.e., transudative and exudative) with the application of Light’s criteria [5]. MPE has been reported in a majority of neoplasms with lung cancer, breast cancer, and lymphomas being the most common causes documented [6].

RCC derived from renal tubular epithelial cells is the most common cause of kidney cancer and has the highest mortality rate of genitourinary malignancies, with an incidence of 2%-4% per year in the US [1]. The risk factors for this condition include cigarette smoking, hypertension, obesity, diabetes, chronic analgesic use, and genetic disorders, such as Von Hippel-Lindau disease and protein polybromo-1 gene [1,7]. Lung metastasis from RCC occurs in 50%-60% of cases, and MPE occurs in 1%-2% of cases [3]. The mechanism is thought to be due to the highly vascular nature of RCC, where arteriovenous fistulas may occur due to its close proximity to the renal vein, inferior vena cava and valveless Batson’s plexus. This may explain the easy hematogenous spread of RCC to the pleura. Other possible mechanisms may include lymphatic spread, although the exact mechanism is unknown [2]. The mere presence of malignant cells in pleural fluid indicates a lower life expectancy, as it is indicative of advanced disease. Prognosis can further be evaluated using the LENT score (pleural fluid lactate dehydrogenase, Eastern Cooperative Oncology Group performance score, neutrophil-to-lymphocyte ratio and tumour type) [8], a measurement of survival in patients with MPE.

The treatment of MPE depends on whether the patient is symptomatic. Asymptomatic patients may be observed until they become symptomatic. If the patient is symptomatic, thoracocentesis may be performed under ultrasound guidance for relief and lung expansion. If the lung does not expand after thoracocentesis, a TPC may be placed, or the patient may be evaluated for loculation. Loculated pleural effusions are most likely associated with pleural inflammation and may involve the production of thick pleura, which causes the persistence of a non-expanding lung, requiring further intervention. For cases were reaccumulation of fluid prevents lung expansion, other interventions may be done. Pleurodesis, the fusion of the parietal and visceral pleura of the lung, obliterates pleural space and prevents reaccumulation. This procedure may be performed using various agents, such as talc, bleomycin, tetracycline, and doxycycline [6,9,10]. In instances where TPC drainage is insufficient, other methods, such as intrapleural tPA use [6,11], are performed to increase the drainage of pleural fluid. Surgical options, such as video-assisted thoracoscopic surgery (VATS), may prove useful if the patient shows no further improvement.

Our case commonly mirrors the typical presentation of most RCCs with flank and back pain. However, our case was unique, as the patient had 2.25 L of aspirate by thoracentesis, with a reaccumulation of 4.3 L over five days. Although thoracentesis is a less invasive method for the diagnosis of MPE, thoracentesis and pleural biopsy were used to confirm the primary source of pleural carcinomatosis causing symptomatic pleural effusion. The diagnosis of RCC is normally confirmed with partial or radical nephrectomy or in some cases renal biopsy as opposed to pleural biopsy, as yield may be low [12]. With such high-volume recurrence, the possibility of high grade renal cell carcinoma [3] must be considered. Afterward, an interventional radiology biopsy of the left-sided renal mass revealed invasive RCC with clear cell features associated with extensive necrosis. An immunohistological evaluation showed that the biopsy was positive for CD10, vimentin, and Pax8 [13,14], which confirmed the diagnosis of renal origin.

Although IR guided biopsy is not often thought to be the standard of treatment, there may be established indications of percutaneous renal mass biopsy as the incidence or renal tumors have increased due to detection by imaging. Though potential risk of rare needle track seeding accounts for 0.01%, complications are also <5% with >90% diagnostic yield [15].

Malignant pleural effusion whether diagnosed by pleural fluid, histological from obtained by biopsy, thoracoscopy or thoracotomy tend to be poor prognosis and usually indicates high grade malignancy. 

## Conclusions

Although metastasis to the lungs is a common presentation, metastatic pleural effusion from RCC pleural carcinomatosis is very rare. In our case, the primary source was confirmed from cytology and immunohistopathology via pleural biopsy, not via renal biopsy or nephrectomy. The high volume of recurrence of pleural effusion over a period of five days may indicate the high grade and severity of renal cell carcinoma. This case showed a unique presentation because the number of cases of the high volume and frequency of the patient's recurrent MPE from RCC has not been well documented in the literature. In a patient with a chronic smoking history and flank pain, clinicians must consider RCC as early detection may provide a more optimal outcome.
